# Isolation of nontuberculous mycobacteria from soil using Middlebrook 7H10 agar with increased malachite green concentration

**DOI:** 10.1186/s13568-017-0373-6

**Published:** 2017-03-23

**Authors:** Yuli Hu, Xinglong Yu, Dun Zhao, Runcheng Li, Yang Liu, Meng Ge, Huican Hu

**Affiliations:** College of Veterinary Medicine, Hunan Agricultural University, No. 1 Nongda Road, Changsha, 410128 Hunan China

**Keywords:** Isolation, Nontuberculous, Mycobacteria, Soil

## Abstract

Environmental exposure is considered to be responsible for nontuberculous mycobacterial infections in humans. To facilitate the isolation of mycobacteria from soil, Middlebrook 7H10 agar was optimized as an enhanced selective medium by increasing the concentration of malachite green. A series of modified Middlebrook 7H10 agar media with malachite green concentrations ranging from 2.5 to 2500 mg/L was evaluated using 20 soil samples decontaminated with 3% sodium dodecyl sulfate plus 2% NaOH for 30 min. Among these modified Middlebrook 7H10 media, the medium with malachite green at a concentration of 250 mg/L, i.e., at the same concentration as in Löwenstein–Jensen medium, was the most effective in terms of the number of plates with mycobacterial growth. This medium was further evaluated with 116 soil samples. The results showed that 87.1% (101/116) of the samples produced mycobacterial growth, and 15 samples (12.9%) produced no mycobacterial growth. Of the plates inoculated with the soil samples, each in duplicate, 5.2% (12/232) showed late contamination. In total, 19 mycobacterial species were isolated, including seven (36.8%) rapidly growing mycobacteria and 12 (63.2%) slowly growing mycobacteria. Our results demonstrate that the modified Middlebrook 7H10 agar with 250 mg/L malachite green is useful for the primary isolation of nontuberculous mycobacteria from soil.

**Electronic supplementary material:**

The online version of this article (doi:10.1186/s13568-017-0373-6) contains supplementary material, which is available to authorized users.

## Introduction

Nontuberculous mycobacteria (NTM) are members of the genus *Mycobacterium*, excluding *Mycobacterium tuberculosis* complex and *Mycobacterium leprae*. *M. tuberculosis* is one of the most important pathogens from this genus. It has been estimated by the World Health Organization that over one-third of the world population is infected with *M. tuberculosis* (Glaziou et al. 2013). Owing to effective measures, the rates of tuberculosis (TB) have gradually declined (Glaziou et al. 2013), whereas those of NTM infections are on the rise in many areas (Brode et al. 2014). Consequently, in some countries with low TB rates, the incidence of NTM infections has been estimated to exceed that of TB (Kotilainen et al. 2011; Brode et al. 2014). Not only are NTM infections risky for individuals with reduced immunocompetence or lung disease (Gopinath and Singh 2010; Brown-Elliott et al. 2012), but there is also increasing evidence showing that the incidence of NTM infections has increased among immunocompetent patients and patients without preexisting lung diseases (Henry et al. 2004; Bodle et al. 2008; Kotilainen et al. 2011). Although only some NTM species (e.g., *Mycobacterium avium*, *Mycobacterium kansasii* and *Mycobacterium fortuitum*) are commonly pathogenic to humans, more than 90 species of NTM have been reported as opportunistic pathogens for humans in two independent investigations (Hoefsloot et al. 2013; van der Werf et al. 2014).

NTM infections are thought to result from exposure to the environment, where NTM species are ubiquitous (Primm et al. 2004), because human-to-human or animal-to-human transmission is rare (Griffith et al. 2007), if any (Ricketts et al. 2014). Soil is the likely source of NTM responsible for human infections, especially in soil-related occupations (e.g., farmers) (Reed et al. 2006; Gopinath and Singh 2010; Hamada et al. 2016). However, recovery of NTM from soil is relatively difficult because NTM attach to soil particles (Falkinham 2002) and the surface soil contains non-mycobacteria at concentrations of approximately 10^8^ cells/cm^3^ (Whitman et al. 1998), which may overgrow in NTM isolation. Several studies have focused on the comparison and optimization of soil decontamination methods (Portaels et al. 1988; Kamala et al. 1994a; Livanainen 1995; Parashar et al. 2004) and the development of media with enhanced selectivity (Ichiyama et al. 1988; Chilima et al. 2006; Narang et al. 2009; Aboagye et al. 2016) for NTM; however, no robust and standardized method for the primary isolation of NTM from soil is yet available.

Middlebrook medium has been widely used in mycobacterial microbiology (Griffith et al. 2007) and can support the growth of most NTM (Wallace et al. 1997). Although Middlebrook medium is more prone to contamination than Löwenstein–Jensen medium (LJ) in the case of soil samples (Livanainen 1995), no significant differences exist in the case of clinical specimens (Somoskövi and Magyar 1999; Idigoras et al. 2000). We noticed that the concentration of malachite green (MG), the only compound providing selective pressure for mycobacterial isolation, is 1000-fold lower in Middlebrook medium than in LJ (0.25 vs. 250 mg/L) (Atlas and Snyder 2006). Thus, we speculated that a modified Middlebrook 7H10 medium with an increased MG concentration may be more effective than LJ as a selective medium for primary isolation of mycobacteria from soil. Therefore, we tested the effectiveness of modified Middlebrook 7H10 media with MG concentrations ranging from 2.5 to 2500 mg/L and further tested an optimized medium for the effectiveness of mycobacterial isolation from 116 soil samples.

## Materials and methods

### Preparation of media

Standard Middlebrook 7H10 (containing 0.25 mg/L MG) was prepared (Atlas and Snyder 2006) and a series of modified Middlebrook 7H10 media was prepared with MG concentrations ranging from 2.5 to 2500 mg/L according to a standard protocol for Middlebrook 7H10 agar preparation (Atlas and Snyder 2006), except that 2% fresh MG (Krieg 1981) was added after the nutrients were autoclaved rather than being mixed with the nutrients before sterilization. The mixed medium was then poured into Petri dishes. PANTA-containing Middlebrook 7H10 agar was prepared similarly, except that MG was replaced with polymyxin B (40,000 U/L)–amphotericin B (4 mg/L)–nalidixic acid (16 mg/L)–trimethoprim (4 mg/L)–azlocillin (4 mg/L) (PANTA; Becton, Dickinson and Company, Spark, USA) reconstituted with oleic acid–albumin–dextrose–catalase (OADC; Becton, Dickinson and Company, Spark, USA) enrichment as recommended by the manufacturer. LJ was prepared according to a standard protocol (Atlas and Snyder 2006). PANTA-containing LJ was prepared similarly to LJ, except that PANTA reconstituted in sterile distilled water was added to the lysed eggs.

### Sample collection

Three sites in Hunan province, China, were chosen for soil sampling, including our campus in Changsha city as well as a community and a park in Zhuzhou city. Soil under trees and herbs at a depth of less than 3 cm was collected, as previously reported (Parashar et al. 2004). At each site, 30–50 soil samples were collected between September 2015 and August 2016. Repeat sampling was not performed within 20 m^2^.

### Sample processing

Sample processing was performed as previously reported (Parashar et al. 2004). Briefly, approximately 5 g of wet soil was transferred into a new 25-mL sterile centrifuge tube and suspended in 15 mL of sterile ddH_2_O. After vigorous shaking for 2 min and standing for 2 min, 1.5 mL of the upper one-third of the turbid supernatants was immediately pipetted into a new sterile 2-mL Eppendorf tube. The suspension was centrifuged at 8000×*g* for 15 min at 4 °C, and the supernatant was discarded. The pellets were resuspended in 1.5 mL of 3% sodium dodecyl sulfate (SDS)–2% NaOH and incubated at room temperature for 30 min. After the incubation, the decontamination solution was removed by centrifugation. The resultant pellets were washed twice with 1.5 mL of sterile phosphate-buffered saline (PBS) and resuspended in 1 mL of sterile PBS for mycobacterial isolation.

### Comparison of selective media

To optimize the concentration of MG in modified Middlebrook 7H10 agar for mycobacterial culture from soil, a two-step assay was performed. Firstly, modified Middlebrook 7H10 agar with MG at the concentration of 2.5, 25, 250, and 2500 mg/L were used for NTM isolation from 20 soils. LJ, PANTA-containing LJ, Middlebrook 7H10 and modified Middlebrook 7H10 with PANTA but without MG media were also inoculated as controls. Each 100-μL decontaminated sample was inoculated onto the media as mentioned above. The media were incubated at 30 °C for 2 months and were examined at 2-day intervals in the first week and weekly thereafter. Secondly, based on the result that modified Middlebrook 7H10 medium with 250 mg/L MG were the most effective, the concentration of MG in modified Middlebrook 7H10 agar with 100 and 500 mg/L MG were tested similarly.

### Application of optimized selective medium

Based on the results obtained by comparison of growth on the selective media, a modified Middlebrook 7H10 medium with 250 mg/L MG was tested with 116 soil samples. Soil used for mycobacterial isolation was treated as described above, and each sample was inoculated onto the modified selective medium in duplicate for culture at 30 and 37 °C, respectively.

### Bacterial identification

To identify mycobacterial species, partial bacterial *rpoB* gene sequences (Adékambi et al. 2003) were amplified by PCR and subsequently sequenced for analysis. Colonies grown on the selective medium were selected based on their growth rate, morphology, and pigmentation and then streaked onto new modified Middlebrook 7H10 medium containing 250 mg/L MG using a calibrated loop to repurify the isolates. After staining cells by the Ziehl–Neelsen method (World Health Organization 1998) to identify acid-fast bacteria, DNA was extracted from the acid-fast bacteria by a heat-shock treatment (96 °C for 15 min) in combination with centrifugation (16,000×*g*, 15 min, 4 °C) as previously described (Radomski et al. 2010). A previously described PCR primer set targeting *rpoB* (Adékambi et al. 2003) was used for PCR and subsequent DNA sequencing. The sequences of the PCR products were determined by a commercial company (Biosune).

### Accession numbers

Partial sequences of the *rpoB* gene of 101 identified mycobacterial strains were deposited in GenBank, with accession numbers from KY400657 to KY400757.

## Results

### Performance of selective media inoculated with decontaminated soil samples

To determine optimal MG concentration in modified Middlebrook 7H10 agar for mycobacteria culture from soil, we first tested MG concentration ranging from 2.5 to 2500 mg/L. Modified Middlebrook 7H10 agar with 25 mg/L MG or lower was insufficient to inhibit nontarget bacteria whereas MG at the concentration of 2500 in the medium can also suppress mycobacteria. By contrast, 65% (13/20) of soils inoculated onto Modified Middlebrook 7H10 agar with 250 mL/L MG produced mycobacterial growth. Notably, the number of plates with mycobacterial growth (13/20) was the same for the media with 250 mg/L MG and PANTA-containing LJ, whereas most of the PANTA-containing LJ showed late heavy contamination (Table 1). However, PANTA-containing LJ provided stronger selectivity in contrast to the modified Middlebrook 7H10 agar with PANTA but without MG. To further optimize the concentration of MG in modified Middlebrook 7H10 agar, we further evaluated modified Middlebrook 7H10 containing 100 and 500 mg/L MG, respectively. The results showed that MG at the concentration of 100 mg/L MG in the medium is as effective as those modified medium with 250 mg/L MG in terms of positive rates and contamination rates. By contrast, modified medium with 500 mg/L MG were sub-optimal because of fewer positive plates. The performance of these 10 selective media were summarized in Table 1.

**Table 1:** Evaluation of 10 selective media for mycobacterial recovery from soil

Media	No. of media		
	Positive	Contaminated	Total
PANTA-containing LJ	13^a^	18	20
LJ	1	19	20
Modified Middlebrook 7H10 with PANTA but without MG	3	17	20
Middlebrook 7H10 with 0.25 mg/L MG	0	20	20
Modified Middlebrook 7H10 with 2.5 mg/L MG	0	20	20
Modified Middlebrook 7H10 with 25 mg/L MG	4	16	20
Modified Middlebrook 7H10 with 100 mg/L MG	13	0	20
Modified Middlebrook 7H10 with 250 mg/L of MG	13	0	20
Modified Middlebrook 7H10 with 500 mg/L of MG	8	0	20
Modified Middlebrook 7H10 with 2500 mg/L of MG	1	0	20

^a^13, 11 of 13 tubes had late contamination

### Isolation of mycobacteria from soil samples

**Fig. 1:**
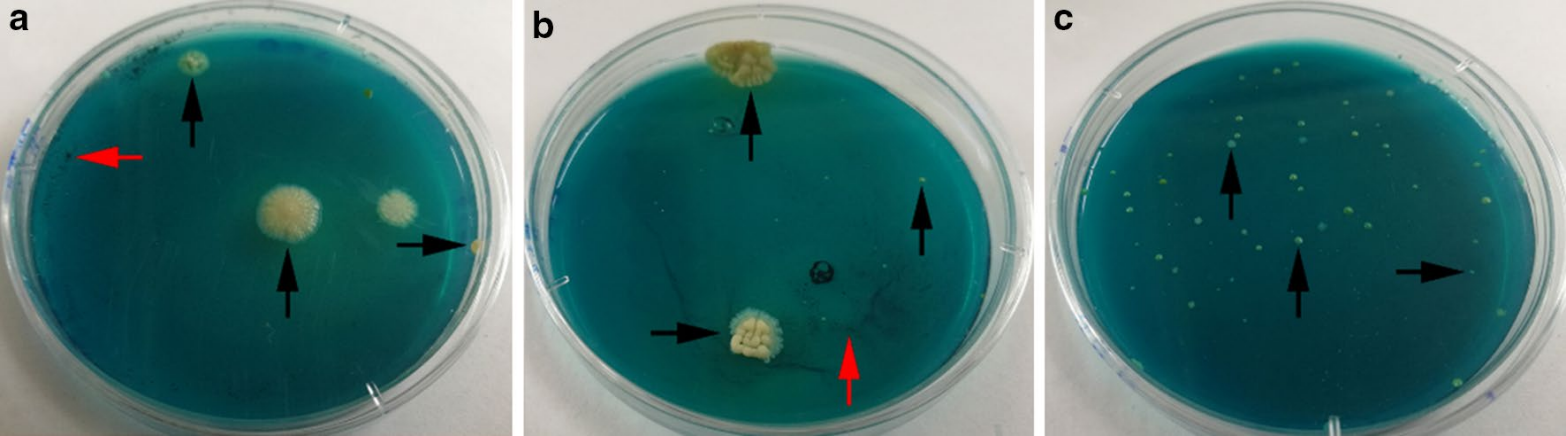
Modified Middlebrook 7H10 medium containing 250 mg/L MG with mycobacteria primarily isolated from soil. **a** Sample collected at campus, **b** sample collected at community, and **c** collected at natural park. *Black arrows* indicate mycobacterial colonies, *red arrows* indicate dried soils

Modified Middlebrook 7H10 medium containing 250 mg/L MG was used to isolate mycobacteria from 116 soil samples. Of these samples, 87.1% (101/116) produced mycobacterial growth, and the remaining showed no growth of microorganisms. Detailed information of soils sampled from the three sites are listed in Table 2 and some of the plates with mycobacterial growth are shown in Fig. 1. Twelve (5.2%) plates showed mycobacterial growth identified by PCR targeting *rpoB* within a month after inoculation, whereas contamination occurred thereafter.

**Table 2:** Details of soil samples isolated in this study

Site	No. of samples	Positive samples	Positive rates (%)
Campus	33	25	75.8
Community	33	28	84.8
Natural park	50	48	96.0
Total	116	101	87.1

### Mycobacterial isolates

To identify the mycobacterial species isolated from the soil samples, 110 isolates were selected based on the bacterial growth rate and morphological characteristics, and their partial sequences of the *rpoB* gene were amplified (Additional file 1: Figure S1) and analyzed after acid**—**fast staining (Additional file 1: Figure S2). Among them, 91.8% (101/110, access number: KY400657 to KY400757) were identified as members of 19 mycobacterial species as shown in Table 3. Nine (8.2%) isolates, whose partial *rpoB* sequence (Additional file 1: Text S1) showed less than 95% similarity with those of known species in GenBank, were not identified according to previously established criteria that intraspecies partial *rpoB* gene shares more than 97% sequence identity (Adékambi et al. 2003, 2006; Adékambi and Drancourt 2004).

**Table 3:** Isolated mycobacterial species

Mycobacterial species	No. of isolates (%)
Rapidly growing	
*M. septicum*	26 (23.6%)
*M. alvei*	10 (9.1%)
*M. fortuitum*	11 (10%)
*M. chelonae*	4 (3.6%)
*M. porcinum*	3 (2.7%)
*M. vaccae*	2 (1.8%)
*M. peregrinum*	1 (0.9%)
Slowly growing	
*M. vulneris*	14 (12.7%)
*M. timonense*	9 (8.2%)
*M. bouchedurhonense*	4 (3.6%)
*M. intracellulare*	3 (2.7%)
*M. mantenii*	3 (2.7%)
*M. colombiense*	2 (1.8%)
*M. farcinogenes*	2 (1.8%)
*M. triplex*	2 (1.8%)
*M. virginiense*	2 (1.8%)
*M. florentinum*	1 (0.9%)
*M. gordonae*	1 (0.9%)
*M. nonchromogenicum*	1 (0.9%)
Unidentified	9 (8.2%)

## Discussion

### Comparison of modified Middlebrook 7H10 agar

Our goal was to optimize a selective medium for primary isolation of NTM from soil. Therefore, a less effective decontamination method (Parashar et al. 2004; Aboagye et al. 2016) including 3% SDS–2% NaOH was used for sample treatment.

Modified Middlebrook 7H10 media with MG concentrations higher than 100 mg/L were acceptable in terms of the contamination rates. However, both the number of plates yielding mycobacteria (Table 1) and the number of colonies on positive plates decreased when the MG concentration was higher than 250 mg/L. A balance between maximizing the activity of mycobacteria and minimizing that of fast-growing microorganisms is important when evaluating methods for mycobacterial recovery (Radomski et al. 2010). Thus, modified Middlebrook 7H10 media with MG at concentrations of 100–250 mg/L were found to be promising for culturing NTM from soil.

### Comparison of LJ, PANTA-containing LJ and modified Middlebrook 7H10 with 250 mg/L MG

The contamination rates in the modified Middlebrook 7H10 medium with the same concentration of MG as in LJ (250 mg/L) were obviously lower than those in LJ (Table 1), indicating that the antimicrobial activity of MG in LJ is diminished because MG can be irreversibly converted to leucomalachite green without bactericidal activity (Jones and Falkinham 2003) by proteins (Duxbury 1993), such as a high concentration of chicken egg albumin (Özer and Çaǧlar 2002). Most of the PANTA-containing LJ was heavily contaminated even though mycobacterial growth was observed before contamination, which is inconsistent with a previous study (Aboagye et al. 2016) showing that only 11 of 139 (7.9%) samples were contaminated. This discrepancy in the results may be attributed to an increased PANTA concentration (2.5-fold), which could enhance the inhibitory effects on nontarget microbes (Peres et al. 2011), and the use of a more effective decontamination method (oxalic acid–NaOH) (Livanainen 1995; Aboagye et al. 2016) in the previous study (Aboagye et al. 2016). The use of different soils may also be a reason.

### Application of modified Middlebrook 7H10 agar with 250 mg/L MG

Considering the diversity of soil microorganisms, a modified Middlebrook 7H10 medium with 250 mg/L MG rather than 100 mg/L MG was used for further isolation of NTM from 116 soil samples. The positive rates of theses samples and the number of mycobacterial species isolated using this medium were equal to or higher than those obtained using other methods based on optimized decontamination procedures and/or enhanced selective medium when applied to more than 100 soil samples (positive rate, 87.1 vs. 18.3–74.7%; number of isolated species, 19 vs. 1–19, respectively) (Kamala et al. 1994b; Donoghue et al. 1997; Chilima et al. 2006; Parashar et al. 2009; Rahbar et al. 2010; Aboagye et al. 2016). These results suggest that Middlebrook 7H10 medium with 250 mg/L MG is useful for primary isolation of NTM from soil.

It is inevitable that some of the mycobacteria are inactivated during mycobacterial isolation because agents including NaOH and MG used in the decontamination step and the selective medium are harmful to mycobacteria (Brooks et al. 1984). In our method, the negative effects of the increased MG concentration on NTM can be partly reduced by the use of a less effective decontamination method.

In conclusion, our data suggest that the modified Middlebrook 7H10 medium with 250 mg/L MG is a useful selective medium for the recovery of mycobacteria from soil. Alternatively, the MG content in Middlebrook 7H10 medium may be adjusted to approximately 100 mg/L when a sample is less complex.

## Additional file

**Additional file 1.** Additional material.

## Abbreviations

NTM: nontuberculous mycobacteria
TB: tuberculosis
LJ: Löwenstein–Jensen medium
MG: malachite green
PANTA: polymyxin B–amphotericin B–nalidixic acid–trimethoprim–azlocillin
OADC: oleic acid–albumin–dextrose–catalase
SDS: sodium dodecyl sulfate
PBS: phosphate-buffered saline
