# Supplementary material for: Isolation of nontuberculous mycobacteria from soil using Middlebrook 7H10 agar with increased malachite green concentration
Source: AMB Express. 2017 Mar 23;7:69. doi: 10.1186/s13568-017-0373-6 (PMC5364124; doi:10.1186/s13568-017-0373-6)
Supplement: Supplementary file 1 — Additional file 1. Additional material. [file 13568_2017_373_MOESM1_ESM.pdf]

Supplementary material

AMB express

Isolation of nontuberculous mycobacteria from soil using Middlebrook 7H10 agar with increased malachite green concentration

Yuli Hu, Xinglong Yu, Dun Zhao, Runcheng Li, Yang Liu, Meng Ge, Huican Hu

College of Veterinary Medicine, Hunan Agricultural University, Changsha 410128, China

Address correspondence to Xing-Long Yu, xlyu999@126.com. Telephone number, +86 731 84673623. Fax, +86 731 84618189.

Figures

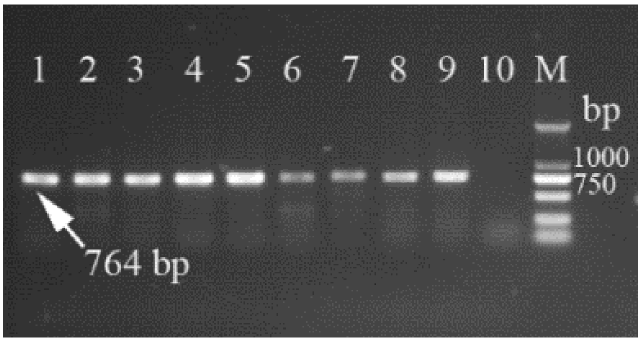

Fig. S1 PCR amplification of partial *rpoB* of 9 unidentified mycobacterial strains. Lane 1, strain 11MF; lane 2, strain 37MF; lane 3, strain B51MF; lane 4, strain A15MF; lane 5, strain B50MF; lane 6, strain C11; lane 7, strain C17; lane 8, strain C31; lane 9, strain M-3; lane 10, negative control; M, DNA marker

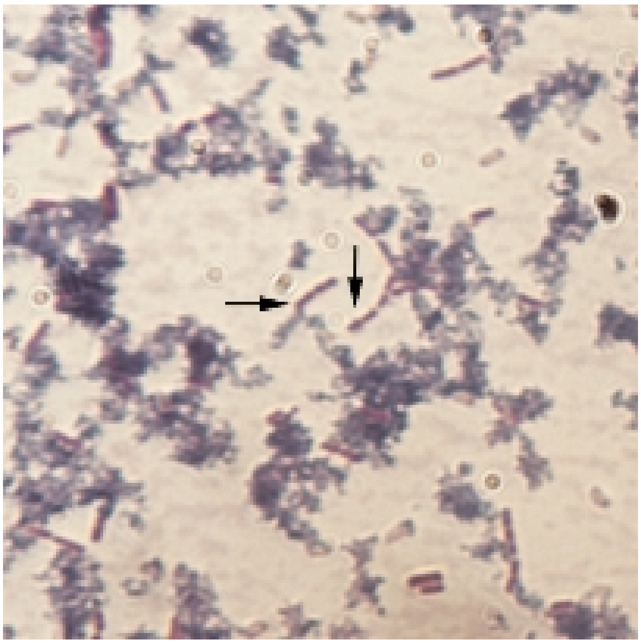

Fig. S2 Acid-fast staining of an isolated mycobacterial strain (×1,000). Black arrows indicate acid-fast bacilli

Text S1

Partial *rpoB* sequence of 9 unidentified mycobacterial strains

>11MF

CGGACGGCTGCTGCGCGCGATCTTCGGTGAGAAGGCCCGCGAGGTCCGCGACACCTCACCTGAAGGTGC

CGCACGGCGAGTCCGGGAAGGTCATCGGCATCCGGGTGTTCTCCCGCGAGGACGATGACGAGCTGCCGG

CCGGGGTCAACGAGCTGGTGCGCGTCTACGTGGCCCAGAAGCGGAAGATCTCCGACGGTGACAAGCTGG
CCGGCCGCCACGGCAACAAGGGCGTGATCGGCAAGATCCTGCCCCAGGAGGACATGCCGTTCCTGCCGG
ACGGCACGCCGGTGGACATCATCCTGAACACCCACGGTGTGCCGCGACGGATGAACATCGGCCAGATCC
TGGAGACCCACCTGGGCTGGATCGCCAAGACCGGATGGAACATCGACGGCTCTGCCGACTGGGCGGAGA
GCCTGCACGAGGACCTCCAGGACCTGCACCCGGACCACATCGAGTCCACCCCCGTGTTCCACGGGTTCA
ACGACGAGGAGGTGAACGGCCTGTTGGCCTGGCTGCTGCCCACCCTCGACGGTGACGACATGGAGGACG
GCGACGGCGGCTACGTGCTGTTCGACGGCCGCAACGGCGACTCGTTCGAGTACCTGGTGACCGTCGGCT
ACGTGTACTTCATGTACCTGCACAACCTGGTGGACGACGAGATCCACGCCATCTCCACCCGCTCGTACT
CAGTTCCCCCCAGAAAAAACCCGGAA

>37MF

CTAGGTCTGCTGCGGGCGATCTTCGGCGAGAAGGCCCGCGAAGTCCGCGACACCTCGCTGAAGGTGCCG
CACGGCGAGTCCGGCAAGGTCATCGGCATCCGGGTGTTCTCCCGTGAGGACGACGACGAGCTGCCCGCC
GGGGTCAACGAGCTGGTTCGGGTCTACGTTGCGCAGAAGCGCAAGATCTCCGATGGTGACAAGCTGGCC
GGCCGCCACGGCAACAAGGGTGTTATCGGCAAGATCCTGCCGGCTGAGGACATGCCGTTCCTTCCGGAC
GGCACCCCGGTCGACATCATCTTGAACACCCACGGTGTGCCGCGACGGATGAACATCGGCCAGATCCTG
GAGACCCACCTGGGCTGGATCGCCAAAACCGGGTGGAACATCGAGCAGGACGGCAACGGCTCCGTTCCG
GACTGGGCTGACAAGCTCCCCGAGGAGCTGTACTCGGCCGGCCCGGACACCCGCACCGCGACTCCGGTG
TTCGACGGCGCGCAGGAGGCCGAGCTGCAGGGCCTGCTGGCCTCGACGCTGGCCAACCGTGACGGCGAG
GTCATGGTCAACGGCGACGGCAAGGCCAAGCTGTTCGACGGCCGCTCCGGAGAGCCGTTCCCGTACCCG
GTGACCGTCGGCTACATGTACATCATGAAGCTGCACCACTTGGTGGACGACAAGATTCACGCCCGTTCC
ACCGGTCCGTATCGGA

>B51MF

GCGTAACGGCTGCTGCGCGCGATCTTCGGCGAGAAGGCGCGCGAGGTCCGCGACACCTCGATGAAGGTG
CCGCACGGCGAGGCCGGCACGGTCATCGGCGTCCGCGTCTTCGACCGCGAGGAGGGTGACGAGCTCGCC
CCGGGTGTGAACCAGCTGGTCCGGGTCTACGTGGCCCAGAAGCGGAAGATCTCCGACGGCGACAAGCTC
GCCGGCCGGCACGGCAACAAGGGCGTGATCTCCAAGATCCTGCCCGTCGAGGACATGCCGTTCCTGGAG
GACGGCACCCCCGTCGACATCGTGCTGAACCCGCTCGGCGTCCCCTCCCGGATGAACGTCGGACAGGTG
ATGGAGCTGCACCTCGGCTGGATCGCCAAGACCGGCTGGGATGTCACCGAGGTCGACGAGCCGTGGGCC

AAGCGCCTGGTGGAGAACGGGATCGGCATGGTTGCCGGTGACCAGCGCCTGGCCACCCCGGTCTTCGAC
GGTGCCACCGAGCAGGAGCTCTCCGGTCTGCTGGAGAACGGTCTGCCGACCGCCGACGGCCTCAAGCTG
GTCGACGGCTCCGGCAAGGCGCGGCTCTTCGACGGCCGCTCCGGCGAGCCCTACCCGGACCCGATCGGT
GTCGGCTACATGTACATGCTGAAGCTGCACCACCTGGTCGACGACAAGATCCACGCCCGCTCCACCGGT
CCGTACTCGTAA

>A15MF

CTAAGCTGCTGCGTGCATCTTCGGTGAGAAGGCCCGCGAGGTTCGCGACACGTCGCTGAAGGTGCCCCA
CGGTGAGTCCGGCAAGGTCATCGGCATCCGCGTGTTCTCGCGTGAGGATGATGACGAGCTGCCCGCCGG
TGTCAACGAGCTGGTCCGCGTCTACGTGGCCCAGAAGCGCAAGATCTCCGACGGCGACAAGCTCGCCGG
ACGCCACGGCAACAAGGGCGTCATCGGCAAGATCCTGCCCGTCGAGGACATGCCGTTCATGCCTGATGG
CACCCCGGTGGACATCATCCTGAACACCCACGGTGTGCCGCGTCGTATGAACATCGGCCAGATCCTGGA
AACCCACCTCGGGTGGGTGGCCAAGGCCGGCTGGAACATCGACGTGGCCGCGGGGACGCCGGAGTGGGC
AGGCAATCTGCCCGAGGGCATGTTGTCTGCGCCGGCCGACAGCATCGTGAGCACCCCGGTGTTCGACGG
TGCCCGCGAAGGGGAGCTGGAGGGCCTGCTCGGCTCGACGCTGCCCAACCGCGACGGTGACGTCATGGT
CAACTCCGAGGGCAAGTCGCAGTTGTTCGATGGCCGCAGTGGCGAACCGTTCCCGTACCCGGTGACGGT
CGGCTACATGTACATCCTCAAGCTGCACCACCTGGTGGACGACAAGATCCACGCTCGCTCCACTGGCCC
GTACTCGTGATCAACCCCCCCAAACAAA

> B50MF

GTTGCGGACGGCTCTGCGTGCGATCTTCGGTGAGAAGCCCGCGAGGTCCGCGACACTTCCCTGAAGGTG
CCGCACGGCGAGTCCGGCAAGGTGATCGGCATCCGGGTGTTCTCCCGCGAGGACGACGACGAACTGCCC
GCCGGCGTCAACGAGCTGGTCCGGGTGTACGTGGCCCAGAAGCGCAAGATCTCCGACGGCGACAAGCTG
GCCGGCCGGCACGGCAACAAGGGCGTCATCGGCAAGATCCTGCCGGTGGAGGACATGCCGTTCCTGCCG
GACGGCACTCCGGTCGACATCATCCTGAACACGCACGGTGTGCCGCGACGGATGAACATCGGCCAGATC
CTGGAAACCCACCTGGGCTGGGTGGCCAAGGCCGGCTGGAACATCGAGGGTGCCCCGGACTGGGCGGCC
AACCTGCCCGAAGAGTTGCGGCACGCCCAGCCGAACCAGATCGTGTCGACTCCGGTGTTCGACGGCGCC
AAGGAAGAGGAGCTGCAAGGCTTGCTTTCGGCCACGCTGCCGAACCGCGACGGCGAGGTGCTGGTCAAC
GAGGACGGCAAGGCGGTGCTCTTCGACGGCCGCAGCGGTGAGCCGTTCCCGTACCCGGTGACCGTCGGC
TACATGTACATCATGAAGCTGCACCACCTGGTGGACGACAAGATCCACGCCCGCTCCACCGGCCCGTAC

TCGATGATCACCCAGCCA

>C11

TAGGCGGGACGCTCTGCGCGCGATCTTCGGTGAGAAGGCCCGCGAGGTCCGCGACACCTCACTGAAGGT

GCCACACGGTGAGTCCGGCAAGGTGATCGGCATTCGCGTGTTCTCCCGCGAGGATGACGACGAACTGCC

CGCCGGTGTCAACGAGCTGGTCCGCGTGTACGTGGCCCAGAAGCGCAAGATCTCCGACGGCGACAAGCT

GGCCGGCCGCCACGGCAACAAGGGTGTCATCGGCAAGATCCTGCCGGTCGAGGACATGCCCTTCATGCC

GGACGGCACCCCGGTCGACATCATCCTGAACACGCACGGTGTGCCGCGACGGATGAACATCGGCCAGAT

CCTGGAAACCCACCTCGGGTGGGTCGCCAAGGCCGGCTGGAAGATCGACGGCAATCAGCTCCCCGAGTG

GGCGTCCAACCTGCCCGAGGATCTGCTGCACGCCGAACCGGATGCGATCGTGTCGACGCCGGTGTTCGA

CGGCGCCAAGGAGGAGGAGCTGCAAGGTCTGCTGTCCTGCACGCTGCCCAACCGCGACGGCGAAGTCAT

GGTGAACGGCGACGGCAAGGCGGTGCTGTTCGACGGCCGCAGCGGCGAGCCGTTCCCGTACCCGGTGAC

CGTTGGCTACATGTACATCATGAAGCTGCACCACCTGGTGGACGACAAGATCCACGCCCGCTCGACCGG

CCCGTACTCGATGTTCCACCCAGCTAA

> C17

TGCTGCGCGCATCTTCGGTGAGAAGGCTCGCGAAGTTCGCGACACCTCGCTCAAGGTTCCGCACGGTGA

GACCGGCAAGGTCATCGGCATCCGCGTCTTCTCGCGTGATGACGACGACGATCTGCCCCCCGGTGTCAA

CGAGCTGGTCCGGGTGTACGTGGCCCAGAAGCGCAAGATCCAGGACGGCGACAAGCTCGCCGGCCGCCA

CGGCAACAAGGGCGTCATCGGCAAGATCCTGCCCGCCGAGGACATGCCCTTCCTGCCTGACGGCACCCC

GGTCGACATCATCCTGAACACCCACGGTGTGCCGCGTCGTATGAACATCGGTCAGATCCTGGAGACCCA

CCTCGGGTGGATCGCCAAGACCGGCTGGAACATCAACGTCGCCGAGGGCGTACCCGAGTGGGCGTCGCG

TCTGCCCGAGGACATGCTCTCGGTCGAGGCCGGCACCAACACCGCGACCCCGGTGTTCGACGGCGCTCG

CGAAGAGGAACTGACGGGCCTGCTGGCCTCGACGCTTCCGAACCGCGACGGCGAGGTGATGGTCAACGC

AGACGGCAAGGCGACCTTGTTCGACGGTCGCAGTGGCGAGCCGTTCCCGTACCCGGTGTCGGTCGGCTA

CATGTACATCATCAAGCTGCATCACTTGGTCGACGACAAGATCCACGCACGCTCGACCGGTCCGTACTC

GATGTCTACCCCAGCC

> C31

CTCTCGTGCATCTTCGGTGAGAAGGCCCGCGAGGTTCGCGACACGTCGCTGAAGGTGCCCCACGGTGAG

TCCGGCAAGGTCATCGGCATCCGCGTGTTCTCGCGTGAGGATGACGACGAGCTGCCCGCCGGTGTCAAC

GAGCTGGTCCGCGTCTACGTGGCCCAGAAGCGCAAGATCTCCGACGGCGACAAGCTCGCCGGACGCCAC
GGCAACAAGGGCGTCATCGGCAAGATCCTGCCCGTCGAGGACATGCCGTTCATGCCTGATGGCACCCCG
GTGGACATCATCCTGAACACCCACGGTGTGCCGCGTCGTATGAACATCGGCCAGATCCTGGAAACCCAC
CTCGGGTGGGTGGCCAAGGCCGGCTGGAACATCGACGTGGCCGCGGGGACGCCGGAGTGGGCAGGCAAT
CTGCCCGAGGGCATGTTGTCTGCGCCGGCCGACAGCATCGTGAGCACCCCGGTGTTCGACGGTGCCCGC
GAAGGGGAGCTGGAGGGCCTGCTCGGCTCGACGCTGCCCAACCGCGACGGTGACGTCATGGTCAACTCC
GAGGGCAAGTCGCAGTTGTTCGATGGCCGCAGTGGCGAACCGTTCCCGTACCCGGTGACGGTCGGCTAC
ATGTACATCCTCAAGCTGCACCACCTGGTGGACGACAAGATCCACGCTCGCTCCACTGGCCCGTACTCG
ATGTTTAACCCAGCA
>M-3
GTCGCGCGATCTTCGGTGAGAAGGCCCGCGAGGTGCGCGACACCTCGCTCAAGGTCCCTCACGGTGAGA
CCGGCACGGTCATCGCGGTCAAGGTCTTCGACAAGGACGACGGCGACGAGCTGCCCCCGGGCGTCAACC
AGCTCGTGCGCGTCTACGTCGCCAACAAGCGCAAGATCACCGACGGCGACAAGCTCGCCGGCCGTCACG
GCAACAAGGGCGTCATCTCCAAGATCCTCCCGGTCGAGGACATGCCCTTCCTCGAGGACGGCACGCCGG
TCGACGTCGTGCTCAACCCGCTCGGTGTCCCCGGTCGAATGAACATCGGCCAGATCCTCGAGCTGCACC
TGGGCTGGGCCGCGAGCCGAGGCTGGGAGATCGAGGGCAACCCCGAGTGGGCCGAGCTCATCCCCGCCG
ACGCCCGCGCCGCCGAGCCGGGCACGCGTGTCGCCTCGCCGGTGTTCGACGGTGTCCGCGAGAACGAGA
TCGTCGGTCTGCTCGACAGCACCCGCAAGACCCGCGACGACGTGCGCCTCATCGACGGCTCCGGCAAGA
CGCACCTGTTCGACGGCCGCTCCGGCGAGCCGTACCCGGAGCCCGTGTCGGTCGGCTACATGTACATCC
TCAAGCTGCACCACCTCGTGGACGACAAGATCCACGCGCGCAGCACGGGTCCGTACTCGATGTTT
